# Identification of valid reference genes for gene expression studies of human stomach cancer by reverse transcription-qPCR

**DOI:** 10.1186/1471-2407-10-240

**Published:** 2010-05-28

**Authors:** Hyun-Wook Rho, Byoung-Chan Lee, Eun-Seok Choi, Il-Ju Choi, Yeon-Su Lee, Sung-Ho Goh

**Affiliations:** 1 Research institute, National Cancer Center, 809 Madu-dong, Goyang, Gyeonggi-do 410-769, Republic of Korea

## Abstract

**Background:**

Reverse transcription quantitative real-time polymerase chain reaction (RT-qPCR) is a powerful method for the analysis of gene expression. Target gene expression levels are usually normalized to a consistently expressed reference gene also known as internal standard, in the same sample. However, much effort has not been expended thus far in the search for reference genes suitable for the study of stomach cancer using RT-qPCR, although selection of optimal reference genes is critical for interpretation of results.

**Methods:**

We assessed the suitability of six possible reference genes, beta-actin (ACTB), glyceraldehydes-3-phosphate dehydrogenase (GAPDH), hypoxanthine phosphoribosyl transferase 1 (HPRT1), beta-2-microglobulin (B2M), ribosomal subunit L29 (RPL29) and 18S ribosomal RNA (18S rRNA) in 20 normal and tumor stomach tissue pairs of stomach cancer patients and 6 stomach cancer cell lines, by RT-qPCR. Employing expression stability analyses using NormFinder and geNorm algorithms we determined the order of performance of these reference genes and their variation values.

**Results:**

This RT-qPCR study showed that there are statistically significant (*p *< 0.05) differences in the expression levels of HPRT1 and 18S rRNA in 'normal-' versus 'tumor stomach tissues'. The stability analyses by geNorm suggest B2M-GAPDH, as best reference gene combination for 'stomach cancer cell lines'; RPL29-HPRT1, for 'all stomach tissues'; and ACTB-18S rRNA, for 'all stomach cell lines and tissues'. NormFinder also identified B2M as the best reference gene for 'stomach cancer cell lines', RPL29-B2M for 'all stomach tissues', and 18S rRNA-ACTB for 'all stomach cell lines and tissues'. The comparisons of normalized expression of the target gene, GPNMB, showed different interpretation of target gene expression depend on best single reference gene or combination.

**Conclusion:**

This study validated RPL29 and RPL29-B2M as the best single reference genes and combination, for RT-qPCR analysis of 'all stomach tissues', and B2M and B2M-GAPDH as the best single reference gene and combination, for 'stomach cancer cell lines'. Use of these validated reference genes should provide more exact interpretation of differential gene expressions at transcription level in stomach cancer.

## Background

Reverse transcription quantitative real-time polymerase chain reaction (RT-qPCR) is a powerful tool for validating the observed gene expression differences, because of its greater sensitivity and specificity. In traditional gene expression studies, a 'reference gene', also called 'internal standard' or 'housekeeping gene' is used for the normalization. The expression of beta-actin (ACTB) and glyceraldehydes-3-phosphate dehydrogenase (GAPDH), used in a majority of studies [1], was reported to vary with experimental conditions [2] and clinical status of the tissue studied (*e.g. *asthma), making these genes unsuitable as internal standards for use in normalization of gene expression [3]. Thus, the validity of the reference gene chosen for statistical analysis is crucial for avoiding the hazard of misinterpreting data and invalid conclusions [4].

It was suggested that at least three considerations should be taken into account in choosing a reference gene: 1) constancy of its expression throughout the intervention, 2) its amplification efficiency and 3) its abundance, which should be similar to that of the genes of interest [5]. In addition, to ensure the relevance, accuracy and correctness of interpretations of RT-qPCR, it is recommended that the precise guidelines for RT-qPCR MIQE (Minimum Information for Publication of Quantitative Real-Time PCR Experiment) should be adhered to [6]. Several tools for statistical analysis such as NormFinder [7], geNorm [8], BestKeeper [9] have been developed to help in the choice of appropriate reference genes. These tools assess the variations in the expression of a number of potential reference genes and suggest which reference gene(s) is appropriate for normalization of gene expression data in a given study.

Stomach cancer is the fourth most common cancer worldwide, with a reported 934,000 cases in 2002 [10]. Survival from stomach cancer is poor since patients are often diagnosed only after the disease has already advanced significantly [11], which makes early detection very important. Screening aiming at early detection involves endoscopic examination. To confirm the presence of cancer, biopsies are taken from suspected tissues and subjected to RT-qPCR to confirm abnormal expression of cancer related genes. But appropriate reference genes have to be identified for valid comparisons between expressions of normal versus cancer genes. Reference genes have been described for RT-qPCR studies in various cancers of other tissues [1,12-21]. However there seems to be no consensus on reference genes for gene expression studies in stomach cancer. We therefore searched PubMed with MeSH terms "gastric cancer", "real-time", and "PCR". In an evaluation of 115 articles published from May 2007 to November 2009, we found that GAPDH (53 cases; 46.1%) and ACTB (41 cases; 35.7%) were the most frequently used reference genes in gastric cancer studies; followed by 18S rRNA (8 cases; 7.0%), beta-2-microglobulin (B2M; 3 cases; 2.6%), hypoxanthine phosphoribosyl transferase 1 (HPRT1; 2cases; 1.7%), TATA binding protein (TBP; 1 case; 0.9%), and beta-tubulin (TUBB; 1 case; 0.9%). In five cases (4.3%), external standard curve was used for absolute quantification (AQ) instead of normalized value by reference gene.

The present study has therefore been designed to find best reference genes for the gene expression studies in stomach cancer. In this study, we investigated the five reference genes that have been most frequently used genes in stomach cancer studies (ACTB, GAPDH, B2M, 18S rRNA, and HPRT1) and for comparison, RPL29, a reference gene used in other cancer studies, in 'non-stomach cancer cell lines', 'stomach cancer cell lines', 'normal stomach tissues' and 'tumor stomach tissues' (Table 1). In order to choose the most appropriate reference gene from the above list, we compared the expressions of glycoprotein NMB (GPNMB), our target gene, with those in the above named list of possible "reference" genes.

**Table 1:** Potential reference genes evaluated in this study.

Gene symbol	GenBank Accession No.	Gene name	Genomic localization	Description
ACTB	NM_001101	Beta-actin	7p15-12	Cytoskeletal structural protein
GAPDH	NM_002046	Glyceraldehyde-3-phosphate dehydrogenase	12p13	Oxidoreductase in glycolysis and gluconeogenesis
HPRT1	NM_000194	Hypoxanthine phosphoribosyl transferase 1	Xq26	Metabolic salvage of purines
B2M	NM_004048	Beta-2-microglobulin	15q21.1	Beta-chain of major histocompatibility complex class I molecules
18S rRNA	NR_003286	18S ribosomal RNA	22p12	Ribosome subunit
RPL29	NM_00992	Ribosomal protein L29	3p21.3-p21.2	Structural constituent of ribosome
GPNMB	NM_001005340	Glycoprotein (transmembrane) nmb	7p15||C	Involved in growth delay and reduction of metastatic potential

## Methods

### Cell lines and human tissues

We obtained cell lines from American Type Culture collection (Manassas, VA, USA) or Korean Cell Line Bank (Seoul, Korea): Six stomach tumor cell lines (SNU-216, SNU-638, SNU-719, AGS, MKN-28 and KATOIII), five non-stomach cancer cell lines (JIMT1, SK-BR-3, SNU-C5, A549, and U87), and two normal human cell lines (HDF, HMEC). All the cell lines were maintained in designated media (Mediatech, Manassas, VA, USA) supplemented with 10% fetal bovine serum (Invitrogen, Calsbard, CA, USA). Twenty matched pairs of normal and tumor stomach tissues were obtained by endoscopic resection during examination of the patients who gave informed consent (Table 2). All procedures were carried out in accordance with protocols approved by institutional review board of National Cancer Center and follow the declaration of Helsinki.

**Table 2:** Features of patients who provided stomach cancer tissues.

		Number of patients
Number of patients	Total	20
	Male	14
	Female	6
		
Age at diagnosis (years)	Range	34-77
	Mean ± SD	60.8 ± 12.1
Disease Stage^†^		
Tumor stage	T1	8
	T2	8
	T3	4
Node stage		
	N0	8
	N1	6
	N2	2
	N3	4

^†^Stage classification follows the TNM classification system by International Union Against Cancer (UICC) [27].

### RNA extraction and cDNA synthesis

Stomach cancer tissue samples were preserved in RNAlater solution (Qiagen, Hilden, Germany) until use for RNA extraction. Total RNA was extracted with TRIzol regent according to the manufacturer's protocol (Invitrogen), and treated with DNaseI on RNeasy Mini column (Qiagen) to remove residual genomic DNA. Concentration and A_260/280 _ratio of purified RNA were measured with Nanodrop ND-1000 (Thermo Scientific, Wilmington, DE, USA), and quality was assessed on Agilent 2100 Bioanalyzer using RNA 6000 Nano kit (Agilent Technologies, Santa Clara, CA, USA). Two μg of poly-dT primed total RNA (random hexamer primed total RNA for 18S rRNA amplification) were reverse-transcribed with Transcriptor Reverse Transcriptase according to the manufacturer's protocol (Roche Applied Science, Mannheim, Germany).

### Reverse transcription quantitative real-time PCR (RT-qPCR)

Based on previous reports, we adopted primers of amplicon length below 200 bp, except for ACTB, to maintain consistency in amplification efficiency (Table 3). The primers for the amplification of GPNMB were designed by Primer 3 software http://frodo.wi.mit.edu/primer3/. We quantified mRNA expression of 6 reference genes and one target gene by RT-qPCR on a Light-Cycler 480 II (Roche Applied Science). RT-qPCR reaction was performed using 5 ng of diluted cDNA, 5 pmole of each primer (Table 3), 5 μl of 2 × Light-Cycler Fast DNA MasterPlus SYBR Green I in final volume of 10 μl. The PCR cycle conditions were set as follows: pre-incubation for 5 minutes at 95°C followed by 45 cycles, with each cycle including 15 seconds at 95°C, 30 seconds at 58°C, and 30 seconds at 72°C. Relative quantification was performed by Light Cycler Software 1.5.0 (Roche Applied Science) based on 'Crossing Point' (Cp) value that defines the cycle number at which the fluorescence signal of the sample exceeds a background fluorescence value.

**Table 3:** Primers for six reference genes and a target gene.

Gene	Forward primer [5'→3'] Reverse sequence [5'→3']	Anchoring Exons	Amplicon size	Spanning on genome	Amplification efficiency	Reference
ACTB	CATCGAGCACGGCATCGTCA TAGCACAGCCTGGATAGCAAC	Exon 3 Exon 4	211 bp	652 bp	1.971	[28]
GAPDH	TGCACCACCAACTGCTTA GGATGCAGGGATGATGTTC	Exon 7 Exon 8	177 bp	370 bp	1.999	[29]
HPRT1	AGACTTTGCTTTCCTTGGTCAG TCAAGGGCATATCCTACAACAA	Exon 6 Exon 8	151 bp	5120 bp	1.949	[30]
B2M	ACTGAATTCACCCCCACTGA CCTCCATGATGCTGCTTACA	Exon 2 Exon 4	114 bp	741 bp	1.924	[28]
18S rRNA	GTAACCCGTTGAACCCCATT CCATCCAATCGGTAGTAGCG	NA^1^	151 bp	151 bp	2.000	[31]
RPL29	GGCGTTGTTGACCCTATTTC GTGTGTGGTGTGGTTCTTGG	Exon 1 Exon 2	120 bp	507 bp	1.937	[16]
GPNMB	TGCGTCCGTGAGAATTCA TGTGCTCCCTCATGTAAGCA	Exon 1 Exon 2	144 bp	6522 bp	1.945	In house design^2^

1. Not available

2. Primers were designed by Primer 3 with human mispriming library screening options.

### Data Analyses

Statistical analyses were performed with GraphPad Prism V4.03 (GraphPad Software, La Jolla, CA, USA). Normality was assessed according to Kolmogorov-Smirnov (KS), D'Agostino-Pearson (DAP), and Shapiro-Wilk (SW) tests. For the distribution of non-normal distributed groups, non-parametric Mann-Whitney U-test and Wilcoxon signed rank test were performed. P-values with *p *< 0.05 were considered statistically significant. We applied NormFinder V12 [7] and geNorm™ V3.44 [8] software to determine the expression values of six candidate reference genes.

## Results

### RNA quality assessment

We assessed the quality of RNA used as starting material in several ways. A_260/280 _ratio measured by Nanodrop was 2.08 ± 0.09 (mean ± SD) confirming that the RNA was pure and protein-free. RNA quality reported as RNA integrity number (RIN) by RNA 6000 Nano Labchip for cultured cell line was 9.7 ± 0.2 (mean ± SD), and 7.4 ± 1.0 for patient tissue samples. For the matched pairs of stomach tissue samples, we did not find any statistically significant difference in either A_260/280 _ratio between normal (2.05 ± 0.03) and tumor (2.04 ± 0.05) tissues (paired Student's *t*-test *p*-value = 0.214) or RIN values between normal (7.2 ± 0.5) and tumor (7.5 ± 1.4) tissue sample groups (paired Student's *t*-test *p*-value = 0.340).

### Expression ranges of candidate reference genes and target gene

**Figure 1:**
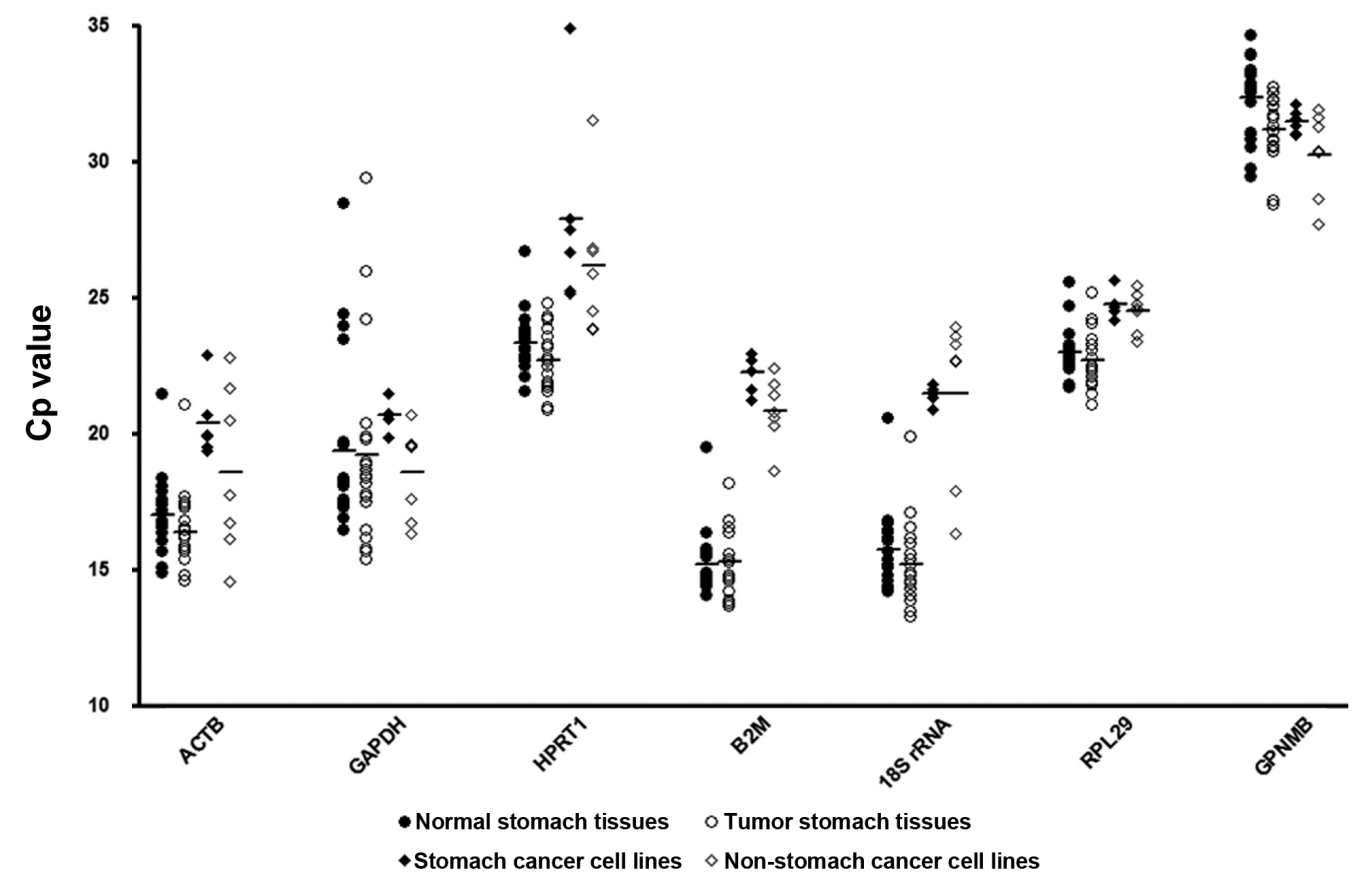
**Expression levels for six candidate reference genes detected by RT-qPCR**. Crossing point (Cp) values in 'stomach cancer cell lines' and 'non-stomach cancer cell lines', 'normal stomach tissues' and 'tumor stomach tissues' are represented as expression level. Horizontal bar in the middle of scattered spots indicates average expression level. The lower the Cp value, the higher is the expression of genes.

We performed RT-qPCR and determined the amplification efficiency of each primer set (Table 3). The expression of six candidate reference genes in terms of Cp values generated from RT-qPCR, are displayed in Figure 1 as scatter plot. The cell lines exhibited a spectrum of Cp values, representing a wide difference in expression, ranging between 14.56 and 34.89, depending on the reference gene used. ACTB and GAPDH showed most abundant expression in both 'stomach cancer cell lines' and 'non-stomach cancer cell lines', but in contrast HPRT1 showed lowest expression level. The expression of target gene GPNMB in Cp values ranged from 27.7 to 32.1 in cell lines. The normality assessment showed HPRT1 in 'stomach cancer cell lines' and 18S rRNA in 'non-stomach cell lines' are not normally distributed by KS-test. Thus, we applied non-parametric Mann-Whitney U-test for comparing non-normal distributed unmatched groups, and it showed significant differences in the expressions of GAPDH (*p *= 0.014) and B2M (*p *= 0.035) between 'stomach cancer cell lines' and 'non-stomach cancer cell lines'.

Human stomach tissues also showed wide variations in Cp values ranging from 13.3 to 29.4 (Figure 1), with highest expression of B2M and 18S rRNA and lowest expression of HPRT1. The expression of target gene GPNMB in Cp ranged from 28.5 to 34.7 in stomach tissues. In normality test, ACTB, HPRT1, and 18S rRNA in 'normal stomach tissues' group and all genes except for GAPDH in 'tumor stomach tissues' followed normal distribution by KS-test. HPRT1, B2M, and RPL29 in tumor stomach tissues passed the normality in DAP- and SW-tests. Thus, for comparing non-normal distributed paired groups, we performed non-parametric Wilcoxon signed rank test. Significant expression increase from normal to tumor stomach tissues (*p *< 0.05) were observed in HPRT1 (*p *= 0.011) and 18S rRNA (*p *= 0.021), but not in ACTB (*p *= 0.058), GAPDH (*p *= 0.918), B2M (*p *= 0.740), or RPL29 (*p *= 0.208).

### Expression stability of candidate reference genes

**Figure 2:**
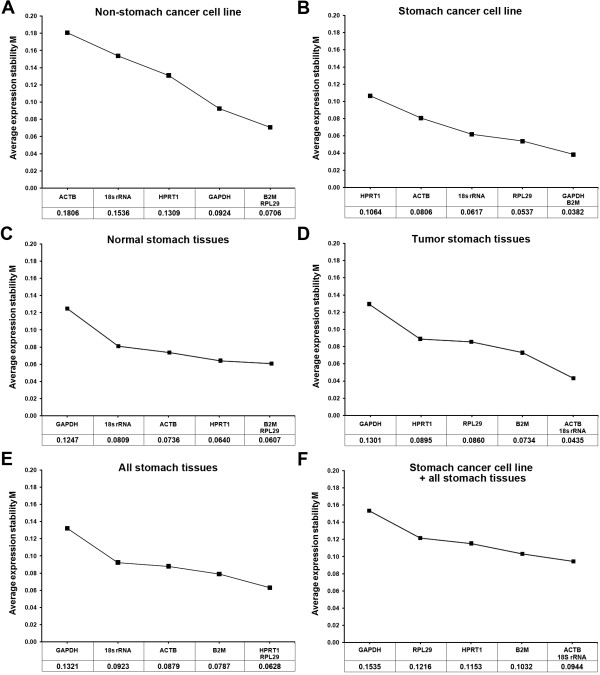
**Average expression stability (M) of six candidate reference genes by geNorm analyses**. Expression stability were plotted in 'non-stomach cancer cell lines' (A), 'stomach cancer cell lines' (B), 'normal stomach tissues' (C), 'tumor stomach tissues' (D), 'all stomach tissues' (E) and 'all stomach cancer cell line and tissues' (F). The least stable reference gene (higher M value) is on the left and the most stable combination (lower M value) is on the right of the plot. Most stable reference genes were deduced by stepwise exclusion of the least stable genes.

**Figure 3:**
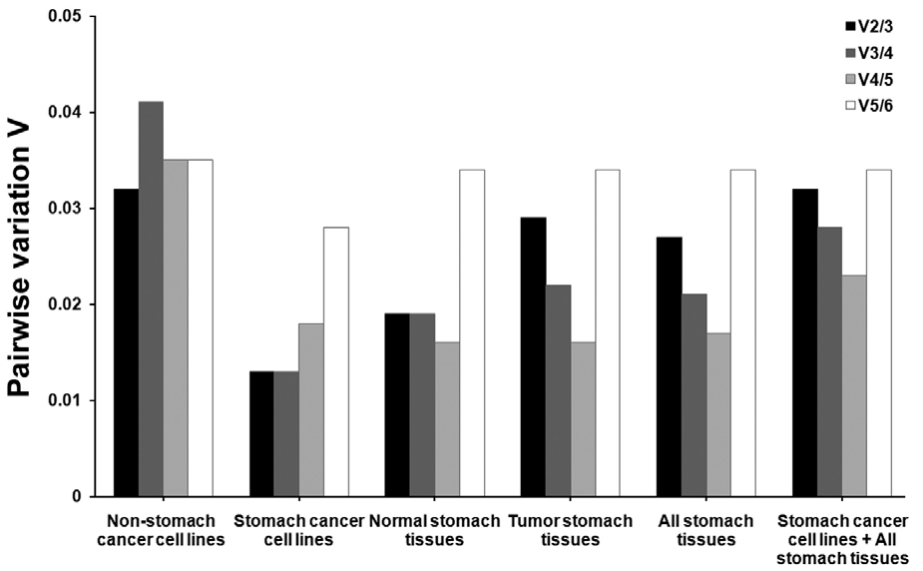
**Pair-wise variation analysis of six candidate reference genes**. Pair-wise variation value (V_n/n+1_) was generated by geNorm analysis. Optimal number of genes was estimated by comparing V_n/n+1_. All the variations were under the default limit of 0.15.

In order to identify the most stable reference genes, we analyzed the expression data with geNorm and NormFinder. We categorized the cell lines and tissues into the following groups - 'non-stomach cancer cell lines', 'stomach cancer cell lines', 'normal stomach tissues', 'tumor stomach tissues', 'all stomach tissues (normal + tumor stomach tissues)' and 'all stomach cancer cell lines and tissues'. The application of geNorm with default limit (M < 1.5) ruled out unstable reference genes and left minimal number of suitable reference genes for each group in the end (Figure 2). To determine the optimal number of genes required for geometric mean normalization, we compared pair-wise variation (V_n_/V_n+1_) calculated by geNorm between each combination of sequential normalization factors (NF_n _and NF_n+1_) for all samples in the group. We applied default threshold (0.15) for cut-off [8] below which inclusion of additional reference genes is not necessary. In all groups evaluated in this study, the pair-wise variations are already below the threshold (Figure 3), thus it was interpreted that using more than two optimal genes are not beneficial to improve accuracy. On the other hand, we found a correlation between variance and slope of the M-value curve. In the case of 'non-stomach cancer cell lines', addition of GAPDH as the third gene to the two optimally expected genes B2M-RPL29 increases by 0.0218 of the stability value M, with its pair-wise variance V_2/3 _of 0.032. In this manner, the coefficient of the correlation between pair-wise variation and the increment of M-value at each interval in this group was determined to be *r*^2 ^= 0.956. For 'stomach cancer cell lines,' the higher V_4/5 _(0.018) and V_5/6 _(0.028) values than V_2/3 _or V_3/4 _explain why the high-scoring HPRT1 and ACTB genes should be excluded. The correlation coefficient was *r*^2 ^= 0.895. The correlation coefficients in 'normal stomach tissues', 'tumor stomach tissues' and 'all stomach tissues' were *r*^2 ^= 0.971, 0.996 and 0.960, respectively. In 'all stomach cancer cell lines and tissues', it showed less correlation (*r*^2 ^= 0.718).

We also applied NormFinder program to the same data sets and calculated stability values. As shown in Table 4, the lowest stability value indicates most stable expression and we ranked genes accordingly. The best single reference gene for each group is as follows; 'non-stomach cancer cell lines' - GAPDH (0.036), 'stomach cancer cell lines' - B2M (0.014), 'normal stomach tissues' - RPL29 (0.028), 'tumor stomach tissues' - RPL29 (0.028), 'all stomach tissues' - RPL29 (0.032) and 'all stomach cell lines and tissues' - ACTB (0.029). The rank of reference genes for 'stomach cancer cell lines' was identical with that from the geNorm analysis, but was slightly different in other categories. NormFinder also estimates the best combination of reference genes by sub-grouping in 'all stomach tissues' - RPL29-B2M (0.005) and in 'all stomach cell lines and tissues' - 18S rRNA-B2M (0.013).

**Table 4:** Ranking of the candidate single reference genes based on their stability values calculated from NormFinder.

Non-stomach cancer cell lines		Stomach cancer cell lines		Normal stomach tissues		Tumor stomach tissues		All stomach tissues		All stomach cell lines + tissues	
**Gene in ranking order**	**Stability value**	**Gene in ranking order**	**Stability value**	**Gene in ranking order**	**Stability value**	**Gene in ranking order**	**Stability value**	**Gene in ranking order**	**Stability value**	**Gene in ranking order**	**Stability value**

GAPDH	0.036	B2M	0.014	RPL29	0.028	RPL29	0.028	RPL29	0.032	ACTB	0.029
RPL29	0.052	GAPDH	0.021	B2M	0.035	B2M	0.039	B2M	0.041	HPRT1	0.038
B2M	0.053	RPL29	0.029	HPRT1	0.038	HPRT1	0.042	HPRT1	0.044	RPL29	0.068
HPRT1	0.110	18S rRNA	0.036	ACTB	0.043	ACTB	0.065	ACTB	0.052	18S rRNA	0.071
18S rRNA	0.112	ACTB	0.060	18S rRNA	0.062	18S rRNA	0.067	18S rRNA	0.055	GAPDH	0.082
ACTB	0.143	HPRT1	0.115	GAPDH	0.140	GAPDH	0.147	GAPDH	0.140	B2M	0.084

### Target gene expression profiles are influenced by reference genes employed for normalization

**Figure 4:**
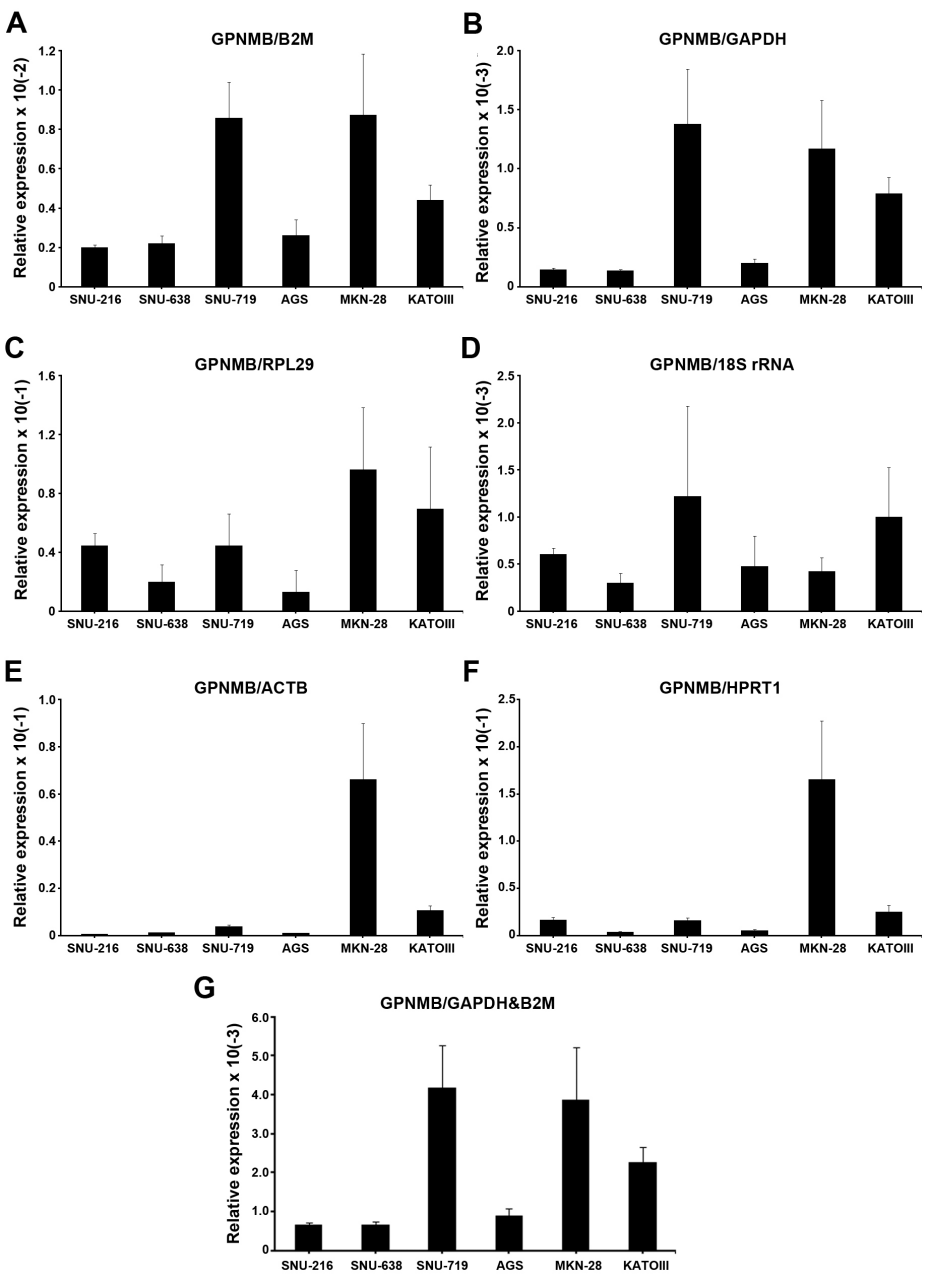
**Relative quantification of GPNMB expression in stomach cancer cell lines depends on different reference genes**. The GPNMB expression in six stomach cancer cell lines were normalized by six single reference genes and best combination derived by geNorm (mean ± SD); normalized by B2M (A), by GAPDH (B), by RPL29 (C), by 18S rRNA (D), by ACTB (E), HPRT1 (F) and geometric mean of B2M-GAPDH combination (G).

For the evaluation of the reference genes in real situation, we chose 'stomach cancer cell lines', 'all stomach tissues' and 'all stomach cell lines and tissues' because cancer researchers' focus on comparing gene expression in normal and tumor tissues as well as stomach originated cancer cell lines for in vitro study. We applied single reference genes and combinations in the relative quantification (RQ) of *GPNMB *as a target gene. GPNMB is a transmembrane glycoprotein and plays a cooperative role with p53 and cytokine-mediated transcription factors in differentiated immune cells [22] and breast cancer [23]. The RQ of GPNMB expression by each six single reference genes and B2M-GAPDH combination in 'stomach cancer cell lines' was compared (Figure 4). The RQs by B2M, GAPDH as single reference gene and B2M-GAPDH that were predicted to be the most optimal combination of reference genes for 'stomach cancer cell lines' by geNorm showed similar high-low patterns (Figure 4A, B and 4G). In comparison, the RQ by RPL29 resulted in an apparently elevated expression in SNU-216 but reduced expression in SNU-719 cell lines (Figure 4C). The RQ by 18S rRNA also showed elevated expression in SNU-216 but lowered expression in MKN-28 (Figure 4D). The RQ by ACTB and HPRT1 showed extremely reduced expression in SNU-719 and KATOIII (Figure 4E and 4F).

**Figure 5:**
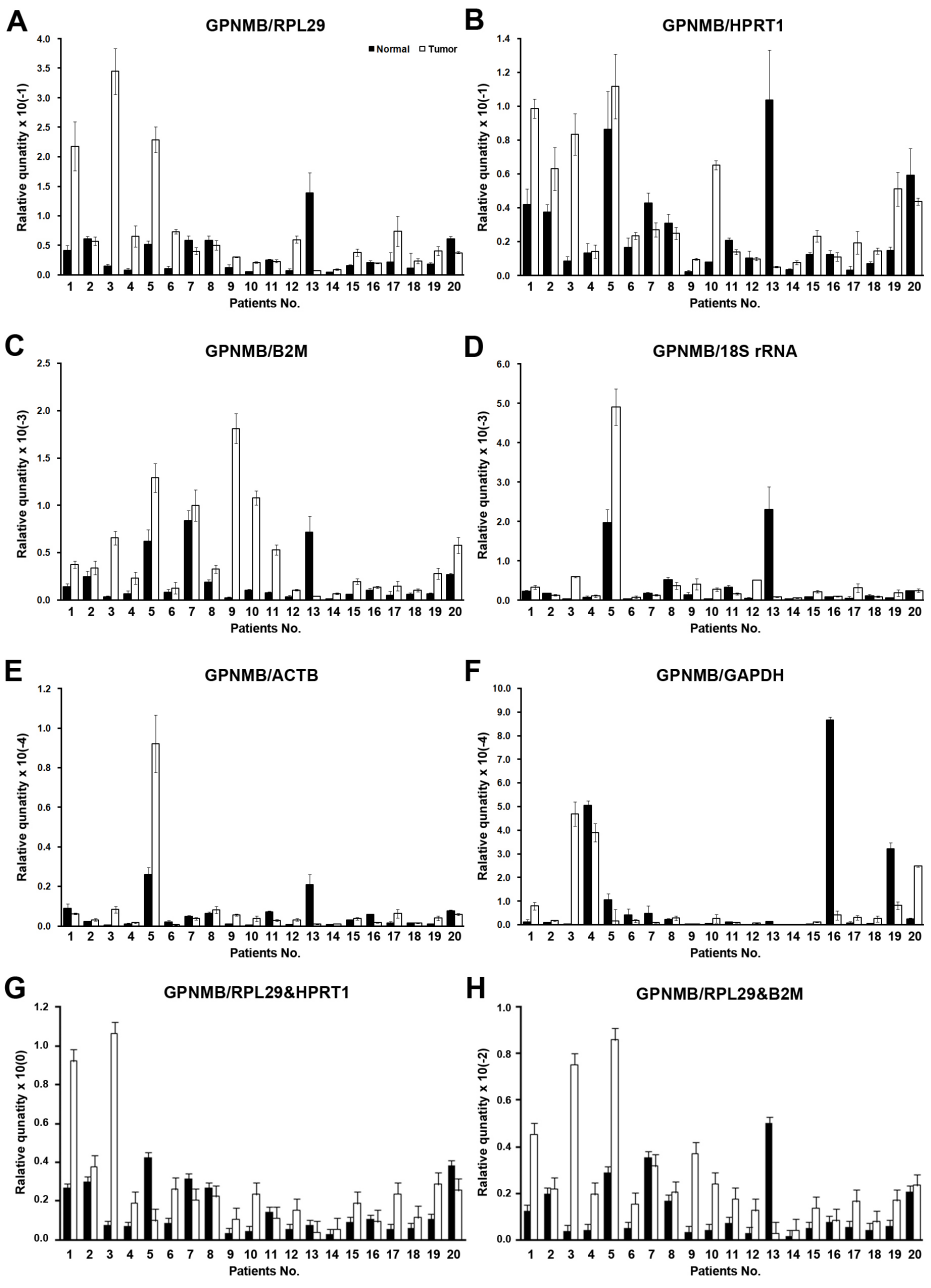
**Relative quantification of GPNMB expression in normal and tumor stomach tissues depends on different reference genes**. The GPNMB expression in stomach cancer tissues (solid bar: Normal, open bar: Tumor) were normalized by six single reference genes and two combinations derived from geNorm and NormFinder (mean ± SD); normalized by RPL29 (A), by HPRT1 (B), by B2M (C), by 18S rRNA (D), by ACTB (E), GAPDH (F), geometric mean of RPL29-HPRT1 (G) and RPL29-B2M (H).

The difference of GPNMB RQ between normal and tumor stomach tissues was patient dependent. RQ is higher in some tumors but opposite in others. The RQ normalized by each six single reference gene did not show the same exact pattern. In case of normalization by RPL29 which was predicted as most stable single reference gene in NormFinder and most stable combination with HPRT1 in geNorm, the high-low pattern of RQ difference between normal and tumor (Figure 5A) was similar to RQ by HPRT1 though there were difference in three patients (Figure 5B). The RQ by B2M (Figure 5c) and 18S rRNA (Figure 5d) showed different pattern from highly ranked single reference genes in more patients. With ACTB (Figure 5E) GAPDH (Figure 5F), the difference became greater; there were differences in 35% of total patients. The RQ normalized by geometric means of RPL29-HPRT1 combination from geNorm (Figure 5G) and RPL29-B2M from NormFinder (Figure 5H) showed similar pattern. When the overall fold change (Tumor/Normal) was compared, RQ of GPNMB by B2M (T/N = 2.46×, paired *t*-test *p *= 0.017) and RPL29-B2M (T/N = 2.08×, *p *= 0.025) showed significant increase from normal to tumor stomach tissues. RQ by RPL29 (T/N = 2.23×, *p *= 0.071), HPRT1 (T/N = 1.34×, *p *= 0.258), ACTB (T/N = 1.60×, *p *= 0.395), 18S rRNA (T/N = 1.36×, *p *= 0.527) and RPL29-HPRT1 (T/N = 1.76×, *p *= 0.086) also showed increasing GPNMB expression in tumor stomach tissues but it was not statistically significant. In comparison, it showed opposite direction of expression difference (T/N = 0.75×, *p *= 0.637) by GAPDH. This suggests that GAPDH expression in tumor stomach tissues are highly elevated compared to the other reference genes. These results also suggest that RQ data of target gene could be interpreted in different ways depending on the reference genes used for normalization.

**Figure 6:**
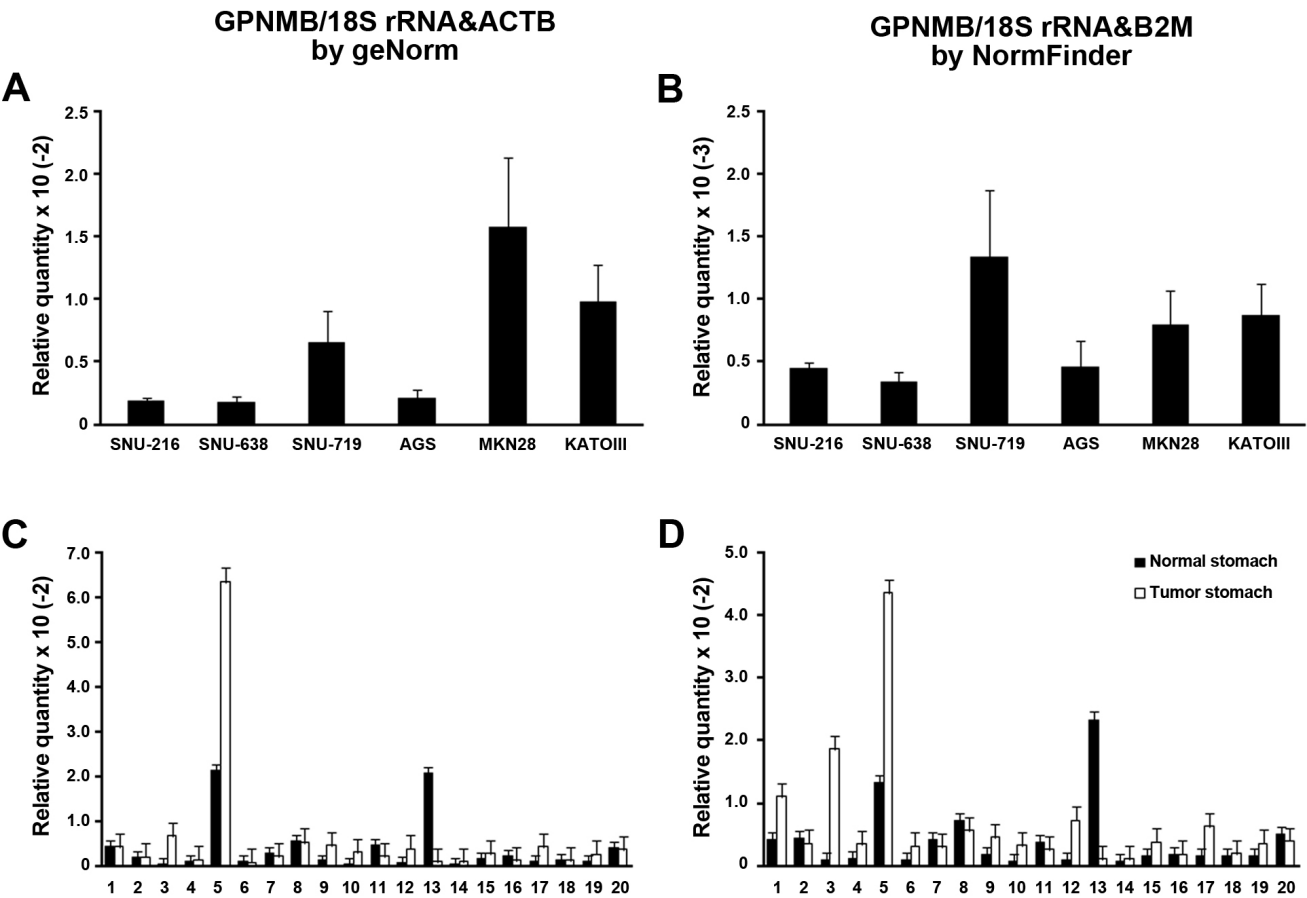
**Relative quantification of GPNMB expression in the pool of all stomach cancer cell lines and tissues depends on different combinations of reference genes**. The GPNMB expression in stomach cancer cell lines and stomach tissues (solid bar: Normal, open bar: Tumor) were normalized by two combinations derived from geNorm and NormFinder (mean ± SD); normalized by 18S rRNA-ACTB in stomach cancer cell lines (A) and stomach tissues (C) and normalized by 18S rRNA-B2M in stomach cancer cell line (B) and stomach tissues (D).

For the 'all stomach cancer cell lines and tissues', NormFinder and geNorm predicted 18S rRNA-B2M and 18S rRNA-ACTB as the best combinations. The pattern of GPNMB RQ by geometric mean of these combinations was similar between them. GPNMB RQs between 'stomach cancer cell lines' and 'all stomach tissues' could be compared within same range with 18S rRNA-ACTB, but there was 1 log of order difference by 18S rRNA-B2M combination. Patterns of RQ in 'stomach cancer cell lines' (Figure 6A, 6B) were similar to RQ by B2M-GAPDH (Figure 4G), but RQ of 'all stomach tissues' by 18S rRNA-ACTB (Figure 6B) were different from (Figure 5G, 5H). It appears that significantly increased 18S rRNA expression in tumor stomach tissues (Figure 1) could contribute to this result. Thus, though these combinations were predicted as best, they are not suitable for the interpretation of data.

## Discussion

Differential gene expression in cancer identified from transcriptome study suggests that some specific genes might be involved in tumorigenesis and metastasis of cancer. RT-qPCR is a robust and specific method for the validation of the identity of candidate genes of stomach cancer, because it detects even very weak signals from extremely small amounts of biopsied samples if the patient is in early stage of cancer. However, in the absence of appropriate reference genes, data obtained are open to question leading to misinterpretation. Prior to this study, no validated reference gene has been identified for 'stomach cancer cell line' or 'stomach cancer tissue', but ACTB and GAPDH have been used most frequently until now without consideration of their inconsistent expressions in different experimental settings and clinical conditions. We examined, in addition to ACTB and GAPDH, four other reference genes, HPRT1, RPL29, 18S rRNA, and B2M that have been evaluated as reference genes in recent studies for other human cancers.

It is evident that choosing appropriate primer set is an important starting point to obtain accurate results. We considered following points in selecting primers. First, we adopted primer sets that were previously reported to have or designed to possess an amplicon length around 200 bp. Second, all of the primer sets were required to span at least two neighboring exons except for 18S rRNA gene which is not an mRNA. The above two points are related to the amplification efficiency. It is necessary that reference gene and target gene maintain similar amplification efficiency [13]. Amplicon length is closely related to amplification efficiency [24]. So one would expect similar efficiency from amplicon of similar length, and higher efficiency from a shorter amplicon. The benefit of shorter amplicon, 70-250 bp, in RT-qPCR is that amplification is "independent" of RNA quality [25]. The amplification efficiency is also affected by gDNA contamination, because competitive binding of primers acts as a limiting factor causing decrease of amplification efficiency [13]. In this context, DNaseI treatment during the RNA purification is crucial to avoid amplification from residual gDNA, but it might not be totally effective. Therefore, our second consideration helps to detect possible contamination with gDNA with different amplicon sizes. We confirmed that each forward and reverse primer is anchored on different exon by BLAT searches on human genome sequences and also that there was no amplified product from contaminating gDNA with extended amplicon length (Table 2). Besides, we also ensured the high quality of RNA, the starting material, in several ways. We also performed the experiments in triplicate for every gene and every sample.

Since the development of qPCR, several statistical programs were developed to identify optimal reference genes. We chose geNorm and NormFinder to analyze the stability of the six reference genes we studied. The geNorm program calculates M-values based on the average pair-wise variation of a particular gene compared with all other studied candidate reference genes and ranks them [8]. In comparison, NormFinder adopts a strategy, called 'model-based approach to estimation of expression variation' [7]. These distinct strategies identified for us the best single or combination reference genes in each group of comparisons. GeNorm identified RPL29-HPRT1 as the most optimal combination for 'all stomach tissues', while NormFinder identified RPL29-B2M instead. Although HPRT1 or B2M was in the best combination identified both by geNorm and NormFinder, respectively, they were ranked third in our single reference gene ranking by each analysis (Table 4). With 'stomach cancer cell lines', the rankings from two analyses were identical, *i.e. *GAPDH-B2M was the most stable reference gene combination followed by RPL29. These results were supported by statistical data, because the highly ranked reference genes have narrower range of variations in expression levels (Figure 1). For example, the most unstable gene, HPRT1, in 'stomach cancer cell lines' has much wider range of expression compared to GAPDH or B2M. This is also true for 'all stomach tissues', since RPL29 and HPRT1 have much narrower range of expression than GAPDH or ACTB.

Some reference genes such as ACTB and B2M are expressed somewhat more in stomach tissues than in cancer cell lines. Cancer cell lines are supposed to be more activated in metabolism, eventually displaying higher transcription activities. However, higher expression of ACTB and B2M was reported in stomach tissues than AGS/SNU-638 stomach cancer cell lines [26] as well as higher B2M expression in liver tissues than HepG2/Hep3B/SK-HEP-1/SNU-182 liver cancer cell lines [17]. In comparison, in this study, the average expression levels of GAPDH and RPL29 were similar in stomach tissues and cancer cell lines. Thus, it appears that metabolically more activated cancer cells are not always in higher transcriptional activity for every gene and every kinds of cell line.

To determine the best reference genes, we analyzed our results with the six candidate reference genes under the suggested rules [5]. First, in terms of amplification efficiency, all primer sets seem acceptable because they have similar and close to perfect amplification efficiency of 2.0 (Table 2). Second, in terms of constant expression in comparable conditions, HPRT1 and 18S rRNA are to be excluded from the best candidate list for comparing gene expression in normal and tumor stomach tissues', because statistical analysis revealed a significant increase in gene expression in tumor tissues compared to normal tissues. Third, in terms of the abundance of reference genes and target gene, ideally the reference should be almost same in its abundance. However, in reality it is hard to find out genes showing exactly same amount of expression. Therefore, it is advisable to use the lesser different reference gene to give out the more accurate interpretation. In agreement with this, RPL29 seems appropriate because the expression is close to that of GPNMB. Lastly, in selecting the best reference genes from two algorithms, we considered whether selecting multiple reference genes in combination is better than selecting a single reference gene alone, because there is still considerable difference of opinion on the use of multiple reference genes as reported in several studies [13]. Since some of the genes included in combination were shown to be differentially expressed between normal and tumor or stomach cancer cell lines and tissues, it is necessary to take into account the consistency of expression ranges of each reference gene. Although RPL29-HPRT1 combination has been suggested as the best for 'all stomach tissues' by geNorm, it is also evident that HPRT1 expression has increased from normal to tumor and this combination is not considered suitable one. In this context, it is not advisable to accept the best combinations for 'all stomach cancer cell lines and tissues'. Both algorithms suggested combinations that have 18S rRNA showing differences in the levels of expression between normal and tumor stomach tissues. Actually, only RPL29 showed consistent expression range for all stomach cancer cells and tissues, suggesting that using single reference gene may be more appropriate for comparisons. Taking these findings together, B2M seems to be the most suitable single reference gene for 'stomach cancer cell lines' and RPL29 for 'all stomach tissues'. RPL29 is also the best for comparing target gene expressions in stomach cancer cells and tissues. Using GAPDH-B2M combination for comparing gene expressions in 'stomach cancer cell lines' and RPL29-B2M combination for comparing in 'all stomach tissues' is therefore recommended. We recognize the limitation of this study in that we examined a limited number of samples, but we feel that our conclusions and recommendations are supported in part by previously deposited expression data from microarray and reports in the literature. In an asthmatic airway study, ACTB and GAPDH were found to be unsuitable as reference genes [4]. Same conclusion was reported for breast, prostate and pancreatic cancers where transcript levels of GAPDH were found elevated [15]. For stomach tissues, we confirmed this in microarray data deposited in ArrayExpress Gene Expression ATLAS http://www.ebi.ac.uk/arrayexpress. The gene expression profile of advance gastric cancer tissues (E-GEOD-2685) showed elevated expression in ACTB (*p*-value = 7.73e-3) and GAPDH (*p*-value = 1.94e-3), but no significant difference with other four candidate reference genes. For the target gene GPNMB, elevation of expression was observed in primary gastric tumors (*p*-value = 1.11e-8; E-GEOD-15460). Thus, it seems clear that blindly choosing just ACTB or GAPDH without such evaluations should be avoided.

## Conclusion

In this study we systematically explored the suitability of potential candidate reference genes for normalization of gene expression in stomach cancer cell lines and tissues. We propose B2M and RPL29 as the best single reference genes for exploring gene expression in 'stomach cell lines' and 'all stomach tissues', respectively. In addition we suggest that GAPDH-B2M combination for normalizing expression in 'stomach cancer cell lines' and RPL29-B2M combination for comparison between normal and tumor in 'all stomach tissues'. RPL29 is also suitable for the comparison in pooled stomach cancer cell lines and tissue samples. The choice of reference genes should depend on the cell lines and/or tissues under study, and there is no single, universal, common optimal reference gene.

## List of abbreviations

RT-qPCR: reverse transcription quantitative real-time polymerase chain reaction; B2M: beta-2-microglobulin; GAPDH: glyceraldehydes-3-phosphate dehydrogenase; HPRT1: hypoxanthine guanine phosphoribosyl transferase 1; ACTB: beta-actin; 18s rRNA: 18S ribosomal RNA; RPL29: ribosomal protein large subunit 29; Cp: crossing point; M: gene stability value; NF: normalization factor; V: variation; RIN: RNA integrity number; RQ: relative quantification; AQ: absolute quantification; KS: Kolmogorov-Smirnov test; DAP: D'Agostino-Pearson test; SW: Shapiro-Wilk test; gDNA: genomic DNA

## Competing interests

The authors declare that they have no competing interests.

## Authors' contributions

H-WR performed all the experiments, statistical analyses, and drafted manuscript. B-CL and E-SC performed RNA purification and performed RT-qPCR experiment. I-JC contributed to the acquisition of patient tissues and clinical data, and also for the interpretation of data. Y-SL participated in designing experiment and interpretation of data. S-HG conceived and design the study and drafted the manuscript. All authors read and agreed to the content of this manuscript.

## Pre-publication history

The pre-publication history for this paper can be accessed here:

http://www.biomedcentral.com/1471-2407/10/240/prepub
